# Dr. George W. Gillette

**Published:** 1905-01

**Authors:** 


Dr. George W. Gillette, of Buffalo, died at his home December 17, 1904, aged 55 years. His death was sudden and due, it is alleged, to some form of disease of the heart. He was born at Cherry Creek, N. Y., in 1849, took the degree of Ph. B., at Cornell University in 1877, and graduated in medicine at the University of Buffalo in 1888. Dr. Gillette abandoned the practice of medicine some years ago and engaged in real estate business, which he pursued until his death. He is survived by his wife and one daughter.

